# Exploring the Application of the Dementia Attitude Scale and Evaluating Its Factor Structure and Stability in Nurse Education

**DOI:** 10.1155/nrp/9845911

**Published:** 2026-02-04

**Authors:** Aoife Conway, Deirdre Harkin, Assumpta Ryan, Paul Slater

**Affiliations:** ^1^ School of Nursing and Paramedic Science, Faculty of Life & Health Sciences, Ulster University, Derry Londonderry Campus, Derry, Northern Ireland, ulster.ac.uk

**Keywords:** dementia, nursing education, person-centred care, positive attitudes, structural validity

## Abstract

**Background:**

The Dementia Attitude Scale (DAS) is one of the most widely used instruments for measuring attitudes towards dementia, yet there is some debate about its factor structure, as deviations from the suggested two‐factor model have been observed and alternative structures have been proposed. This study aimed to analyse and compare the different factor structures of the DAS and examine the consistency of these structures over time.

**Methods:**

A cross‐sectional series design was used to collect longitudinal data from a census of nursing students over a 2‐year period. The DAS was administered at three time points (Time Point 1 [baseline]: *n* = 247; Time Point 2 [Year 1]: *n* = 239; Time Point 3 [Year 2]: *n* = 216). Descriptive statistics were calculated using SPSS, and structural equation modelling was performed in JASP to assess structural validity. Confirmatory factor analysis was conducted on the DAS and on each alternative structure proposed at each time point to evaluate fit and stability.

**Results:**

The findings indicated that the two‐factor model demonstrated the best fit and stability across all time points, suggesting it is the most effective model for capturing the DAS constructs within this population. Reliability analysis showed that the two‐factor model maintained strong internal consistency for ‘dementia knowledge’ and ‘social comfort’.

**Conclusion:**

The DAS provides valuable insights into the effectiveness of educational interventions, enabling educators to identify and address areas where students may require additional support. This study highlights the need for future research to explore the translation of these shifts into clinical behaviours, as well as the cultural and contextual factors influencing attitudes towards dementia.

## 1. Introduction

Currently, over 55 million people are living with dementia, and this figure is projected to nearly double every 20 years, reaching 78 million by 2030 and 139 million by 2050 [1]. In health care, the growing number of individuals living with dementia increases the demand for nurses who are not only clinically skilled but also compassionate in their care for both people with dementia and their carers. High‐quality nursing care is crucial for addressing the complex needs of people living with dementia and enhancing the quality of life for them and their families or carers [2–4]. However, effective dementia care requires more than clinical competence and knowledge, and it is also shaped by attitudes towards dementia.

Alzheimer’s Society United Kingdom is calling on governments in England, Wales and Northern Ireland to establish a statutory requirement for all registered care providers to receive dementia education [5]. Similarly, the World Health Organization and Alzheimer’s Disease International have highlighted the importance of dementia education for preregistration nurses [6, 7]. Higher education institutions play a crucial role in meeting these needs, equipping preregistration nurses with the skills and attitudes necessary to deliver high‐quality dementia care and enhancing the capacity of the future dementia care workforce [8–10].

Breckler describes an attitude as a combination of emotional, cognitive and behavioural responses to a person, object or situation [11]. These responses impact the care provided, nurses’ perceptions of their roles and their motivation to acquire and apply new skills [12–15]. For nursing students, developing positive attitudes towards dementia and individuals living with this condition is of the utmost importance. Students who hold favourable attitudes are more likely to engage empathetically with patients, adopt person‐centred care approaches and provide high‐quality care to those living with dementia [16]. These attitudes, often shaped during their training, can be maintained over time and have a lasting influence on future practice [15, 17–20]. Consequently, educational programmes that foster positive attitudes in nursing students are crucial in preparing the next generation of nurses for the increasing demands of dementia care. Such an emphasis on attitude development not only benefits healthcare recipients but also equips students with the emotional and cognitive skills needed to succeed in demanding healthcare environments [5].

Measuring attitudes accurately is essential for understanding and addressing existing gaps in education and training. By understanding the attitudes held by students, educators can tailor programmes to foster empathy, reduce stigma and promote a deeper commitment to person‐centred care [21, 22]. Therefore, psychometrically sound instruments that measure these attitudes over an extended period (e.g., a 3‐year undergraduate degree programme) offer insights critical for curriculum development and ongoing education, ultimately strengthening the healthcare workforce’s preparedness for dementia care.

## 2. Literature Review

Selecting an instrument to evaluate attitudes towards dementia requires thoughtful consideration. The choice should be informed by the specific characteristics and objectives of the programme or intervention. Important factors to consider include the target audience (e.g., formal or informal caregivers), the psychometric robustness of the instrument, alignment between the instrument’s domains and programme content, cultural relevance and readability in relation to the literacy levels of the target population [23]. On reviewing the literature, most studies that explored attitudes towards dementia adopted a quantitative approach to measurement [19, 22], using scales specifically designed to measure attitudes towards dementia, such as the Dementia Attitude Scale (DAS) (see Table 1) and the Attitude towards Dementia Scale [44, 45]. Other studies used tools to assess specific behaviours associated with dementia‐related attitudes, for example, the Attitudes towards Lying to People with Dementia (ALPD) questionnaire [46, 47]. Additionally, custom open‐ended questionnaires tailored to assess attitudes towards specific interventions have been developed by some researchers [48, 49]. Additionally, some studies use qualitative approaches, such as interviews and focus groups [50–52].

**Table 1:** Studies using the Dementia Attitude Scale (DAS) across different populations of nursing students globally.

Study	Country	Setting	Population	Factor structure
Sunaryo et al. [24]	Indonesia	Indonesia	*N* = 334 nursing students	Two‐factor model—‘dementia knowledge’ and ‘social comfort’ as identified by O’Connor and McFadden [54]
Aljezawi et al. [25]	Jordan	English	*N* = 275 nursing students	Two‐factor model—‘dementia knowledge’ and ‘social comfort’ as identified by O’Connor and McFadden [54]
Aljezawi et al. [26]	Jordan	English	*N* = 118 nursing students	Two‐factor model—‘dementia knowledge’ and ‘social comfort’ as identified by O’Connor and McFadden [54]
Campbell et al. [27]	United States	English	*N* = 163 nursing students	Unidimensional use
Çetİnkaya et al. [28]	Turkey	Turkish	*N* = 131 medical students *N* = 195 nursing students	Three‐factor structure: ‘Supporting attitude’, ‘Accepting attitude’ and ‘Exclusionary attitude’
Chang et al. [29]	Korea	English	*N* = 295 nursing students	Two‐factor model—‘dementia knowledge’ and ‘social comfort’ as identified by O’Connor and McFadden [54]
Hebditch et al. [30]	England	English	*N* = 488 nursing students	Unidimensional use
Khatiwada et al. [31]	Nepal	English	*N* = 177 nursing students	Two‐factor model—‘dementia knowledge’ and ‘social comfort’ as identified by O’Connor and McFadden [54]
Kimzey et al. [32]	America	English	*N* = 94 nursing students	Two‐factor model—‘dementia knowledge’ and ‘social comfort’ as identified by O’Connor and McFadden [54]
Kimzey et al. [33]	America	English	*N* = 108 nursing students	Two‐factor model—‘dementia knowledge’ and ‘social comfort’ as identified by O’Connor and McFadden [54]
Korkmaz et al. [34]	Turkey	Turkish	*N* = 784 nursing students	Three‐factor structure: ‘Supporting attitude’, ‘Accepting attitude’ and ‘Exclusionary attitude’ as identified by Çetinkaya et al. [28]
Lokon et al. [35]	United States	English	*N* = 130 students from other disciplines *N* = 26 nursing students	Two‐factor model—‘dementia knowledge’ and ‘social comfort’ as identified by O’Connor and McFadden [54]
Maharaj [36]	United States	English	*N* = 76 nursing students	Unidimensional use
Mastel‐Smith et al. [37]	United States	English	*N* = 19 nursing students *N* = 13 occupational therapy students	Unidimensional use
Mastel‐Smith et al. [38]	United States	English	*N* = 100 nursing students	Unidimensional use
Poreddi et al. [39]	India	English	*N* = 122 nursing students	Two‐factor model—‘dementia knowledge’ and ‘social comfort’ as identified by O’Connor and McFadden [54]
Scerri and Scerri [40]	Spain	English	*N* = 280 nursing students	Two‐factor model—‘dementia knowledge’ and ‘social comfort’ as identified by O’Connor and McFadden [54]
Seltmann and Teichmann [41]	Germany	German	*N*= nursing students	Unidimensional use
Torrence et al. [42]	United States	English	*N* = 18 nursing students *N* = 23 other students	Unidimensional use
Vasquez et al. [43]	United States	England	*N* = 76 nursing students	Unidimensional use

The DAS is an instrument designed to measure attitudes, grounded in the widely accepted tripartite model of attitude. This model conceptualises attitude as the willingness of an individual, based on experience, to respond in a particular way to a person, situation or idea, reflected across cognitive (assumptions and beliefs), affective (feelings and emotions) and behavioural (actions) domains [53]. Developed by O’Connor and McFadden [54], the DAS was created in response to the shortcomings of existing instruments that measured attitudes towards dementia. Previous tools were often limited in scope, lacking validation across diverse samples and failing to capture the full complexity of attitudes towards dementia. These instruments did not provide robust evidence for convergent and divergent validity and lacked a general scale that could be applied broadly. The DAS was therefore developed to fill these gaps, offering a more comprehensive and psychometrically sound measure of attitudes towards dementia [54]. The DAS was originally developed as a two‐factor model and aimed to capture both the affective and behavioural aspects of attitudes towards dementia through a ‘comfort’ subdomain and the cognitive aspect through a ‘knowledge’ subdomain. The reported psychometric properties of the DAS are strong, comparing favourably with similar scales that measure attitudes towards ageism and disabilities [54]. This instrument has been utilised across a diverse range of participants, including healthcare professionals [55, 56], medical and nursing students [40] and informal carers [57, 58]. At the time of writing this paper, the DAS had been cited in 175 papers. To ensure alignment with our study and to narrow the focus, we will concentrate specifically on undergraduate (preregistration) nursing students.

Table 1 summarises findings from various studies that used the DAS across different populations of nursing students globally. Table 1 highlights some variability in the factor structures identified across studies. Although some studies reported a two‐factor model, several studies treated the scale as functionally unidimensional, and a smaller number of studies identified a three‐factor model. All papers that implemented a two‐factor model did so based on the framework identified by O’Connor and McFadden [54], which includes the factors ‘dementia knowledge’ and ‘social comfort’. Similarly, studies that utilised a three‐factor model adhered to the structure established by Çetİnkaya et al. [28], comprising the factors ‘supporting attitude’, ‘accepting attitude’ and ‘exclusionary attitude’. This study did not aim to statistically compare findings with previous research but focused on evaluating the internal structure of DAS within the context of this specific educational setting by applying factor structures suggested within the literature.

### 2.1. Research Aim

The aim of this study was to examine and confirm the structural validity of the DAS as a tool to measure attitudes towards dementia care among a cohort of preregistration nursing students in a region of the United Kingdom over a 2‐year period. This was achieved by applying factor structures proposed in the existing literature within the context of a single educational setting.

## 3. Objectives

1. To compare different factor structures within the DAS (e.g., one‐factor, two‐factor and three‐factor models) across multiple time points to determine which structure provides the best fit.
2. To assess the stability of model fit for each factor structure over time, evaluating the reliability of the DAS in capturing attitudes towards dementia care as nursing students progress through their programme.

## 4. Methods

### 4.1. Study Design

This study forms part of a larger research project that uses longitudinal methods to evaluate the Dementia Education Programme (DEP) [59, 60]. At Ulster University, the DEP is a mandatory part of the adult and mental health nursing curricula, designed to prepare nurses with the skills, knowledge and confidence needed for delivering high‐quality dementia care. The DEP is structured as a multilevel curriculum that provides a progressively deeper understanding of dementia, aiming to enhance students’ knowledge, attitudes and confidence in dementia care. The programme takes a multimodal approach to education, delivered by educators with extensive expertise in dementia care and in teaching methodologies. Furthermore, it integrates interprofessional collaborations with a local healthcare trust and voluntary organisations, fostering the sharing of expertise and community‐based insights. The DEP is grounded in the principles of the Person‐Centred Nursing Framework [61], ensuring a holistic and evidence‐based approach to dementia education.

Full ethical approval was granted for this study (Committee Application Number: XXXXX).

### 4.2. Participants

This study was conducted at a U.K. university with an annual intake of adult and mental health nursing students to an accredited undergraduate programme. All students who met the inclusion criteria were invited to participate in the study (must be a preregistration nursing student at the participating university undertaking the BSc Hons Nursing Degree in Adult or Mental Health 2022 intake). At the time of recruitment, 430 students were enrolled in these programmes at Point 1, 413 at Point 2 and 374 at Point 3. All students were provided with information on the study and invited to participate.

### 4.3. Data Collection

A data collection instrument was developed to assess participants’ attitudes towards dementia using the DAS alongside demographic questions. Data were collected at three distinct time points [54]:

1. Time Point 1 (Baseline): Before any dementia‐related education, at the start of the degree programme
2. Time Point 2 (Year 1): After the completion of the first level of the DEP
3. Time Point 3 (Year 2): After the completion of the second level of the DEP

Data were collected through the online platform JISC. All eligible and willing participants were provided with access to the data collection instrument via a weblink and QR code delivered by email. All items required a response for completion, meaning that the response‐to‐item ratio was equal to the total number of participants.

### 4.4. DAS

The open‐source DAS is a 20‐item instrument. The items are rated on a 7‐point Likert scale. Responses range from strongly disagree (1) to strongly agree (7), and six items on the scale are reverse‐scored. Higher scores indicate more positive attitudes. Two‐factor structure, originally identified by the scale developers O’Connor and McFadden [54], includes the factors ‘dementia knowledge’ and ‘social comfort’. However, a one‐factor structure (incorporating all items) and a three‐factor structure, established by Çetİnkaya et al. [28], comprising the factors ‘supporting attitude’, ‘accepting attitude’ and ‘exclusionary attitude’, have also been applied. To the authors’ knowledge, no previous studies have compared these three‐factor structures.

### 4.5. Data Analysis

Data were collected using longitudinal methods, with each participant assigned a unique identifier to allow tracking of individual changes over time. For psychometric testing, each time point was analysed separately as cross‐sectional data. Although the longitudinal design holds significant potential for understanding how attitudes evolve or are maintained over time, the cross‐sectional analysis was essential for validating the scale’s internal consistency and reliability across participants at multiple time points. The analysis was conducted using SPSS (version 29) for descriptive statistics and JASP for structural equation modelling (SEM). Descriptive statistics and measures of dispersion were calculated for all items to inform subsequent analyses. To assess normality, values for skewness and kurtosis between −2 and +2 were considered acceptable for establishing univariate normal distribution [62]. Diagonally weighted least squares (DWLS) estimation was applied due to the values for skewness and kurtosis. DWLS tends to provide more accurate parameter estimates and model fit that are more robust to the type of variables and non‐normality, particularly when the data do not meet the assumption of multivariate normality. Compared to maximum likelihood (ML), DWLS is often preferred when dealing with non‐normal data, as it reduces bias in parameter estimates and improves fit when the data are not normally distributed [63, 64].

Confirmatory factor analysis (CFA) was conducted on three datasets to verify the factor structure of the DAS and assess the model’s validity over time. All three proposed models—one‐factor, two‐factor and three‐factor structures—identified in Table 1 were applied to each of the datasets. The consistent factors used for this study are the two‐factor model identified by O’Connor and McFadden [54], which includes the factors ‘dementia knowledge’ and ‘social comfort’, and the three‐factor model established by Çetİnkaya et al. [28], comprising the factors ‘supporting attitude’, ‘accepting attitude’ and ‘exclusionary attitude’. This approach enabled a comparison of the fit and stability of each model across different time points, providing an insight and understanding into the most robust structure for measuring attitudes toward dementia among nursing students over an extended period. To evaluate the model’s fit, several indices were calculated, including the chi‐square test, goodness‐of‐fit index (GFI), Tucker–Lewis index (TLI), comparative fit index (CFI), root‐mean‐square error of approximation (RMSEA) and standardised root‐mean‐square residual (SRMR). Acceptable fit statistics were set at GFI: > 0.95, TLI: > 0.90, CFI: > 0.95, RMSEA: < 0.08 and SRMR: < 0.08 [65–67].

### 4.6. Reporting

The Consensus‐Based Standards for the Selection of Health Measurement Instruments (COSMIN) guidelines and STROBE checklist of items that should be included in reports of cross‐sectional studies were used as structured frameworks to report the psychometric properties of the DAS and ensure that all critical aspects of the study were thoroughly documented [68], facilitating transparency and clarity in the research process [69]. Mokkink et al. [70] noted that studies on measurement properties often lack key information, preventing readers such as clinicians, scientists and funders from fully understanding the methods, results and implications of the research for patient‐reported outcome measures. Enhancing the reporting of these studies increases transparency, clarifies the risk of bias and highlights their relevance to scientific knowledge. By adhering to the COSMIN and STROBE reporting guidelines, this paper aims to provide clear insights into all aspects of the study.

## 5. Results

### 5.1. Sample Characteristics

Table 2 reports the characteristics of the participants across three time points, revealing a slight decrease in total participants over time. The response‐to‐item ratio was 28:1, 26:1 and 24:1 across the three time points, respectively.

**Table 2:** Participant characteristics.

	Time Point 1	Time Point 2	Time Point 3
Total participants	249	237	216

Age	18–25 = 182 (73.15%) 25–35 = 38 (15.3%) 35–45 = 24 (9.6%) 45–55 = 5 (2%)	18–25 = 174 (73.4%) 25–35 = 39 (16.5%) 35–45 = 21 (8.9%) 45–55 = 3 (1.3%)	18–25 = 147 (68.1%) 25–35 = 42 (19.4%) 35–45 = 23 (10.6%) 45–55 = 4 (1.9%)

Course	Adult nursing = 191 (76.7%) Mental health nursing = 58 (23.3%)	Adult nursing = 198 (83.5%) Mental health nursing = 39 (16.5%)	Adult nursing = 173 (80.1%) Mental health nursing = 43 (19.3%)

Gender	Female (including trans women) = 225 (90.4%) Male (including trans men) = 20 (8%) Nonbinary = 1 (0.4%) Prefer not to say = 3 (1.2%)	Female (including trans women) = 218 (92%) Male (including trans men) = 17 (7.2%) Nonbinary = 1 (0.4%) Prefer not to say = 1 (0.4%)	Female (including trans women) = 195 (90.3%) Male (including trans men) = 19 (8.8%) Nonbinary = 1 (0.5%) Prefer not to say = 1 (0.5%)

Ethnic group	Asian or Asian British (Indian, Pakistani, Bangladeshi, Chinese, any other Asian background) = 3 (1.25%) Black, Black British, Caribbean or African (Caribbean, African, any other Black, Black British or Caribbean background) = 5 (2.0%) Mixed or multiple ethnic groups (White and Black Caribbean, White and Black African, White and Asian, any other mixed) or = 4 (1.6%) White (English, Welsh, Scottish, Northern Irish, British, Irish, Gypsy, Irish Traveller, Roma, any other White background) = 237 (95.2%) Prefer not to say = 1 (0.4%)	Asian or Asian British (Indian, Pakistani, Bangladeshi, Chinese, any other Asian background) = 3 (1.3%) Black, Black British, Caribbean or African (Caribbean, African, any other Black, Black British or Caribbean background) = 5 (2.1%) Mixed or multiple ethnic groups (White and Black Caribbean, White and Black African, White and Asian, any other mixed) or = 2 (0.8%) White (English, Welsh, Scottish, Northern Irish, British, Irish, Gypsy, Irish Traveller, Roma, any other White background) = 227 (95.8%)	Asian or Asian British (Indian, Pakistani, Bangladeshi, Chinese, any other Asian background) = 4 (1.9%) Black, Black British, Caribbean or African (Caribbean, African, any other Black, Black British or Caribbean background) = 3 (1.4%) Mixed or multiple ethnic groups (White and Black Caribbean, White and Black African, White and Asian, any other mixed) or = 1 (0.5%) White (English, Welsh, Scottish, Northern Irish, British, Irish, Gypsy, Irish Traveller, Roma, any other White background) = 206 (95.4%) Prefer not to say = 2 (1%)

### 5.2. Measures of Distribution

Table 3 displays the mean values, standard deviations (SDs), skewness and kurtosis for each statement across three time points. Values of skewness and kurtosis often exceed the accepted range of −2 to +2, suggesting that these items do not meet the assumptions of univariate normality.

**Table 3:** Measures of skewness and kurtosis.

	Time Point 1 (baseline)			Time Point 2 (Year 1)			Time Point 3 (Year 2)		
	Mean (SD)	Skewness	Kurtosis	Mean (SD)	Skewness	Kurtosis	Mean (SD)	Skewness	Kurtosis
Difficult behaviours may be a form of communication for people with ADRD^∗^	6.41 (0.96)	−1.47	1.26	6.65 (0.82)	−2.58	6.63	6.60 (0.83)	−2.29	4.81
It is rewarding to work with people who have ADRD	6.39 (1.10)	−1.81	2.63	6.68 (0.72)	−2.20	3.78	6.60 (0.91)	−2.97	9.86
I am afraid of people with ADRD.	6.45 (1.08)	−2.51	6.47	6.50 (1.18)	−2.96	8.96	6.59 (0.98)	−3.44	14.45
People with ADRD can be creative	5.90 (1.28)	−1.08	0.88	6.46 (1.05)	−2.43	7.01	6.61 (0.77)	−2.09	3.72
I feel confident around people with ADRD	5.35 (1.55)	−0.89	0.43	6.24 (1.04)	−1.85	5.06	6.39 (0.75)	−1.32	1.77
I am comfortable touching people with ADRD	5.71 (1.37)	−1.04	0.99	6.42 (0.97)	−2.27	7.54	6.45 (0.90)	−1.87	3.71
I feel uncomfortable being around people with ADRD	5.67 (1.82)	−1.41	0.83	5.68 (2.11)	−1.44	0.47	5.32 (0.83)	−1.00	−0.77
Every person with ADRD has different needs	6.79 (0.61)	−3.04	8.66	6.88 (0.51)	−4.82	25.30	6.90 (0.39)	−4.78	24.88
I am not very familiar with ADRD	4.59 (2.04)	−0.42	−1.10	5.58 (1.69)	−1.15	0.35	5.79 (1.59)	−1.54	1.56
I would avoid an agitated person with ADRD	5.39 (1.69)	−0.87	−0.13	5.95 (1.40)	−1.45	1.66	6.08 (1.21)	−1.47	1.67
People with ADRD like having familiar things nearby	6.49 (0.96)	−2.06	4.22	6.78 (0.67)	−4.40	26.42	6.73 (0.62)	−3.09	11.30
It is important to know the history of people with ADRD	6.47 (0.98)	−2.25	6.10	6.87 (0.59)	−6.48	51.98	6.78 (0.53)	−2.73	8.15
It is possible to enjoy interacting with people with ADRD	6.43 (1.30)	−2.50	5.34	6.50 (1.50)	−3.10	8.28	6.74 (0.72)	−3.04	8.44
I feel relaxed around people with ADRD	5.01 (1.48)	−0.36	−0.34	5.64 (1.39)	−1.01	0.84	5.85 (1.18)	−0.92	0.14
People with ADRD can enjoy life	6.59 (0.87)	−2.58	7.51	6.81 (0.70)	−4.73	25.01	6.88 (0.41)	−3.99	17.73
People with ADRD can feel when others are kind to them	6.59 (0.88)	−2.10	3.50	6.87 (0.53)	−4.53	21.821	6.88 (0.47)	−5.11	31.23
I feel frustrated because I do not know how to help people with ADRD	3.92 (2.01)	0.16	−1.18	4.95 (1.83)	−0.49	−0.954	5.17 (1.68)	−0.73	−0.50
I cannot imagine caring for someone with ADRD	6.35 (1.21)	−2.25	5.05	6.45 (1.19)	−2.71	7.399	6.51 (1.03)	−2.61	7.28
I admire the coping skills of people with ADRD	6.57 (0.90)	−2.10	3.60	−2.71 (0.16)	−3.75	15.308	6.87 (0.43)	−4.14	20.10
We can do a lot now to improve the lives of people with ADRD	6.76 (0.69)	−4.01	21.95	6.87 (0.48)	−4.45	23.272	6.86 (0.50)	−4.31	19.83

^∗^Alzheimer’s disease and related dementia (ADRD).

### 5.3. CFA

The findings within Table 4 provide an analysis of the model fit and the stability of one‐, two‐ and three‐factor models when assessing data from three time points: baseline (T1), Year 1 (T2) and Year 2 (T3). The chi‐square statistic (chi‐square, degrees of freedom ratio along with its *p*‐value), the CFI, TLI, GFI, RMSEA and SRMR values were evaluated at each time point. The two‐factor model consistently performs best, with higher CFI and TLI values and lower RMSEA and SRMR scores at each stage, and is a stronger model fit with stability over the 2‐year period compared to the one‐ and three‐factor models. The chi‐square statistic also confirms that the two‐factor model provides the best overall fit across the three time points (T1, T2 and T3), particularly at T2 and T3, where the chi‐square values are lower and the *p*‐values are nonsignificant, suggesting an acceptable model fit.

**Table 4:** Model fit indices for one‐factor, two‐factor and three‐factor models across three time points.

	One‐factor model			Two‐factor model			Three‐factor model		
	T1	T2	T3	T1	T2	T3	T1	T2	T3
Chi‐square (*χ* ^2^, D.f, *p*)	360.29, 170 *p* < 0.01	287.0, 170 *p* < 0.001	203.68, 170, *p* = 0.040	199.95, 169, *p* = 0.052	190.30, 169, *p* = 0.125	112.43, 169, *p* = 1.0	328.10, 167 *p* < 0.001	246.98, 167, *p* < 0.001	198.88, 167, *p* = 0.046
CFI	0.86	0.79	0.95	0.98	0.96	1.00	0.88	0.85	0.96
TLI	0.84	0.76	0.95	0.97	0.96	1.08	0.86	0.83	0.95
GFI	0.89	0.82	0.88	0.93	0.88	0.93	0.90	0.85	0.89
RMSEA	0.07	0.05	0.03	0.03	0.02	0.00	0.06	0.05	0.03
SRMR	0.10	0.13	0.11	0.08	0.08	0.07	0.10	0.11	0.11

The one‐ and three‐factor models displayed mixed stability. The one‐factor model, with CFI and TLI values initially below acceptable fit thresholds, showed improvement by T3. However, the higher RMSEA values, fluctuating GFI and higher chi‐square values with significant *p*‐values at all time points, indicate that this model is less robust and less efficient in capturing nuanced aspects within the data across multiple time points. Similarly, the three‐factor model demonstrated limited stability, as seen in its moderate fit indices, although it did reach acceptable fit by T3. These trends suggest that although the one‐ and three‐factor models can achieve acceptable fit in certain contexts, they lack the stability and robustness provided by the two‐factor structure over extended time frames. The two‐factor model demonstrated both high initial fit and sustained stability, with model fit values improving or remaining adequate by Time Point 3. This stability of fit suggests that the two‐factor model remains suitable and may even improve in representing the data’s underlying structure as familiarity or stability in the responses develops over time (potentially due to increased learning).

### 5.4. Reliability

Table 5 highlights the measure of internal consistency and reliability, indicating how well the items within a model measure the same construct. The one‐factor model shows stable and moderate reliability across time points. The two‐factor model maintains relatively strong reliability for ‘dementia knowledge’, but ‘social comfort’ fluctuates. The three‐factor model has weaker and less stable reliability, particularly for ‘accepting attitude’ and ‘exclusionary attitude’, suggesting potential issues with the internal consistency of these factors.

**Table 5:** Coefficient *α* of the one‐factor, two‐factor and three‐factor models across three time points.

Factor structure	Domain	T1 coefficient *α*	T2 coefficient *α*	T3 coefficient *α*
One‐factor model		0.74	0.72	0.77

Two‐factor model	Dementia knowledge	0.77	0.72	0.70
	Social comfort	0.74	0.64	0.78

Three‐factor model	Supportive attitude	0.65	0.57	0.62
	Acceptive attitude	0.62	0.39	0.62
	Exclusionary attitude	0.80	0.64	0.45

## 6. Discussion

The findings within this paper offer a significant contribution by affirming the robustness of the two‐factor model of the DAS, establishing its stability and confirming the DAS as a reliable tool for measuring attitudinal changes in nursing students over time, offering educators insights into how students’ attitudes towards dementia evolve throughout their education. By tracking these changes longitudinally, HEIs can assess the impact of their educational interventions, ensuring that students are progressively developing the positive attitudes needed to provide high‐quality care to those living with dementia and their carers. This is particularly critical in dementia care, where fostering empathy, respect and understanding is integral to delivering effective and compassionate care [20]. The application of the DAS across a degree programme allows educators to identify specific points where attitudes may stagnate or regress. This insight can inform the design of targeted interventions to reinforce positive attitudes at key stages, bridging gaps in knowledge or experience that might otherwise impact the students’ development. It also provides educators with the opportunity to tailor their teaching strategies to better support students’ learning and attitude development. Although the results presented in this paper pertain to nursing students, the two‐factor structure has been used and confirmed with other healthcare students, healthcare staff and informal carers [54, 71–73].

Pedagogical strategies such as experiential learning [51], workshops [46] and simulation exercises have proven effective in influencing attitudes [52, 74]. However, despite their potential, there is a dearth of evidence regarding the long‐term impact of these methods. It is unclear which interventions result in the most meaningful and enduring changes in attitudes [22]. Although these educational interventions may shift attitudes during the educational programme, effectiveness in sustaining changes once students enter clinical practice has not been fully explored [22]. Although cognitive and affective shifts are essential components of attitudinal change, the ultimate goal of nurse education is to translate these shifts into behaviours that enhance patient care. Within the research, there is a widely acknowledged consensus that changes in attitudes are closely tied to changes in behavioural intentions, which often serve as precursors to actual behaviours [75, 76]. Behavioural intention, recognised as the strongest predictor of behaviour [75, 76], is influenced by attitudes and other psychological factors such as subjective norms and self‐efficacy [77]. To predict specific behaviours, such as shifts in approaches to dementia care, it is crucial to examine attitudes directly associated with that behaviour. It is believed that these attitudes can significantly shape an individual’s intention to act, which, in turn, drives actual behaviour [77]. This highlights the importance of employing a rigorous and robust tool like the DAS to effectively measure these critical attitudes and monitor attitudinal changes over time. However, it is equally vital to assess the practical translation of these attitudinal shifts into specific behaviours within dementia care. Without such evaluation, educators are left to assume that improved attitudes inherently lead to better behaviours—an assumption that, despite its importance, remains largely under‐researched. This knowledge gap highlights the need for longitudinal studies that track the trajectory of attitudinal changes from education into clinical practice.

Alongside the pedagogical strategies previously mentioned that can impact attitudes towards dementia, some studies highlight the concept of the ‘hidden curriculum’. The hidden curriculum comprises values, norms and beliefs, among other elements, that are not formally documented or part of the official teaching but are conveyed to students through informal and implicit cues and which can significantly influence their attitudes towards dementia [78–80]. These messages are often conveyed through interactions with faculty, peers and clinical mentors, shaping students’ perceptions and behaviours in ways that may not align with the formal curriculum. Although quantitative tools such as the DAS are valuable for measuring attitudinal changes, additional attention is required to explore the underlying contributing mechanisms for these changes (both positive and negative). Quantitative attitudinal research typically identifies correlations (e.g., between an educational programme and improved attitudes), rather than establishing causation. To address this limitation, educators may consider integrating qualitative methods, such as reflective journals, focus groups and participatory approaches, into their evaluation frameworks. These qualitative methods may offer deeper insights into the factors influencing students’ attitudes, thereby providing a more comprehensive and holistic understanding of their learning experiences.

Cultural differences significantly influence attitudes towards dementia, which vary widely across cultural contexts [81]. In some cultures, dementia may be perceived as a natural part of ageing, whereas in others, it may carry significant stigma or misconceptions. These varying attitudes shape how dementia is managed and understood, both within families and healthcare systems [82]. Although the DAS has been validated across diverse populations, its factor structures differ possibly due to the cultural norms towards dementia care, and this suggests the need for culturally sensitive adaptations to educational tools and interventions. This is essential to prepare the future workforce to deliver care to culturally diverse population groups. Integrating education on cultural differences in dementia care into the curriculum can help students develop the knowledge and skills needed to provide care that respects the unique values, beliefs and practices of individuals and families impacted by dementia [82].

## 7. Conclusion

This study provides robust evidence supporting the two‐factor model of the DAS as the most stable and reliable framework for assessing attitudes towards dementia across multiple time points. The two‐factor model outperformed both the one‐ and three‐factor models in terms of fit indices, demonstrating consistent validity and reliability over an extended period. These findings highlight the potential of the DAS as a longitudinal tool to evaluate nursing students’ attitudinal development throughout their education. The stability of the two‐factor model reinforces its suitability for tracking attitudinal shifts, offering educators a valuable resource to evaluate the impact of dementia education interventions. By using the DAS to monitor these changes, educators can identify areas where students may require additional support, enabling targeted and effective interventions. Although the DAS provides a rigorous quantitative measure of attitudinal changes, future research should explore the translation of these shifts into behaviours within clinical practice, ensuring that improved attitudes lead to meaningful and compassionate care practices.

## 8. Strengths and Limitations

This study has several notable strengths. The extended design allowed for data collection across multiple time points, providing valuable insights into the stability of the DAS in measuring attitudes over a 2‐year period and enhancing the robustness of the findings. The inclusion of a diverse and sizeable cohort of nursing students increases the generalisability of the results within the context of nursing education. Additionally, the methodological rigour employed throughout the study increases confidence in the scale’s ability to measure attitudes accurately. However, some limitations should be acknowledged. Although the DAS is a validated tool, it may not comprehensively capture all dimensions of attitudes towards dementia, particularly behavioural intentions. Using complementary instruments could have provided a more holistic understanding of attitudes and their potential impact on practice. Furthermore, the study did not explicitly explore cultural or contextual differences among participants such as age, gender, cultural background and previous experience with dementia, although these factors may influence the interpretation of the scale and the broader applicability of the findings. Future studies may wish to undertake formal comparative analyses to explore similarities and differences in factor structures across contexts. Future research could address these gaps to build on the strengths of this study.

## Disclosure

All authors gave final approval of the version to be published and agreed to be accountable for all aspects of the work. All authors who contributed significantly to the work are listed. No other authors were involved in this work.

## Conflicts of Interest

The authors declare no conflicts of interest.

## Author Contributions

Aoife Conway: conceptualisation, methodology, formal analysis, investigation, data curation, writing–original draft, writing–reviewing and editing, visualisation and project administration. Dr Deirdre Harkin: conceptualisation, validation and writing–reviewing and editing. Prof Assumpta Ryan: conceptualisation, validation and writing–reviewing and editing. Dr Paul Slater: conceptualisation, methodology, formal analysis, writing–reviewing and editing and visualisation.

All authors made substantial contributions to the conception or design of the work, as well as the acquisition, analysis and interpretation of data. They were involved in drafting the work or reviewing it critically for important intellectual content.

## Funding

This research received no funding.

## Data Availability

The data that support the findings of this study are available on request from the corresponding author. The data are not publicly available due to privacy or ethical restrictions.
